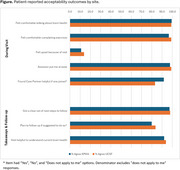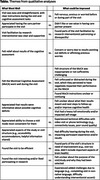# Patient acceptability of brain health screening in primary care: results from the electronic health record Risk of Alzheimer's and Dementia Assessment Rule (eRADAR) pragmatic clinical trial

**DOI:** 10.1002/alz70858_101260

**Published:** 2025-12-25

**Authors:** Mikael Anne Greenwood‐Hickman, Clarissa W Hsu, Sascha Dublin, Alana Elop, Tyler D Barrett, Judith Walsh, Sherry Yam, Leonardo Colemon, Lawrence Madziwa, Leah Karliner, Deborah E Barnes

**Affiliations:** ^1^ Kaiser Permanente Washington Health Research Institute, Seattle, WA, USA; ^2^ University of California, San Francisco, San Francisco, CA, USA

## Abstract

**Background:**

Approximately half of people with dementia are undiagnosed, yet little is known about patient responses to real‐world screening for dementia in primary care. As part of a pragmatic trial, we evaluated patient‐reported acceptability and satisfaction with a primary care‐based, targeted cognitive screening visit.

**Method:**

Study sites were Kaiser Permanente Washington (KPWA) and University of California, San Francisco (UCSF) healthcare systems. Participants identified as high‐risk for having undiagnosed dementia were invited for a “brain health” visit that assessed independence in daily activities, depressive symptoms and cognitive impairment. Participants were encouraged to include a care partner at the visit. Afterward, participants were given a post‐visit evaluation survey that included structured and open‐ended questions about visit satisfaction. Using a mixed methods approach, proportions of “agree” responses were calculated for structured questions, and free text responses were analyzed using Rapid Group Analysis Process (Rap‐GAP).

**Result:**

*N* = 426 (*n* = 327/64.6% KPWA, *n* = 99/50.3% UCSF) completed a post‐visit survey. KPWA survey respondents were 43% female, 91% Non‐Hispanic White, and 71% had a college degree or higher; UCSF respondents were 52% female, 66% Non‐Hispanic White, and 76% had a college degree or higher. Based on the visit, 13% of respondents were referred to their PCP for follow‐up regarding memory concerns. From structured questions (Figure), >90% of participants reported they felt comfortable discussing brain health and found the visit helpful for understanding their current brain health. Qualitative themes (Table) confirmed these positive findings, but also surfaced some important critiques. Participant concerns included not understanding the next steps recommended for cognitive evaluation, highlighting the need to share next steps clearly and, when appropriate, to share summary recommendations with a care partner directly. Few participants reported feeling upset by the visit (∼11% of participants). Qualitative themes suggested that when upset occurred, reasons included concern about their results, perceptions that the assessment of their functioning might be inaccurate due to hearing loss or difficulty understanding the instructions, or finding questions about their daily functioning patronizing.

**Conclusion:**

High‐risk patients participating in primary care‐based cognitive screening visits found them acceptable. Targeted screening approaches could provide a supportive pathway to improve dementia detection in primary care.